# Intravenous Injection of Milk in Bright’s Disease

**Published:** 1879-07

**Authors:** John Bryson

**Affiliations:** St. Louis Courier of Medicine


### Intravenous Injection of Milk in Chronic Bright's Disease--with Remarks.

By John Bryson, M. D., St. Louis Courier of Medicine.

Case.—I first saw Mrs.———, æt about thirty-five, in August, 1878. She had already suffered from chronic renal disorder for several months, and was pale, weak, waxy-colored, and cachetic. The entire body, below the breasts, was highly œdematous, but I could make out no accumulation of fluid in the peritoneal sac. The area of precordial dullness was not increased, and, in fact, there was nothing abnormal about the heart. The urine, I afterwards determined, ranged in quantity from eighteen to thirty ounces in twenty-four hours; was pale, of a specific gravity of 1004 to 1011, and contained albumen to the extent of from one-half to two grains to the ounce. It deposited an abundant sediment, made up principally of fragments of epithelial cells, but containing, at the same time, a very few hyaline and pale casts. Several times there appeared, also, some large, waxy casts; but only once a granular cast. There was no evidence of an acute commencement of the renal disorder, but on the contrary, the history distinctly indicated that it was chronic from the beginning.

No uræmic symptoms had appeared. I believed the case to be one of *chronic parenchymatous* nephritis (large, white kidney.)

Arsenic materially lessened the amount of albumen in the urine; but hot-air baths and iron did not improve the general condition. Finally, acupuncture removed the œdema, in a short time, but after its disappearance the patient was not much better, except that she could be moved about and had more ease. Emaciation was extreme, the dry, yellow and branny skin appearing to be drawn over a mere skeleton. Digestion was almost *nil*, and the impoverished condition of the blood (hypalbuminosis) was marked.

On September 27th, at 8 o'clock, A. M., the patient appearing to be in a dying condition, about three ounces of milk, fresh from the cow, and of neutral reaction, were injected into the median cephalic vein of the left arm.* Immediately, on the milk beginning to flow into the vein, the patient had an uncomfortable, sinking sensation, was slightly nauseated, and soon lost consciousness. The pulse became slower, weaker, and eventually imperceptible at the wrist, but gradually came up, and at the end of the operation was 60 per minute. At 8:40 the pulse was 82, and there were slight chilly sensations. At 10:30, pulse was 118, and reaction was complete. At 2:45 P. M., pulse 108, skin moist, and patient had some appetite; took more than a glassfull of milk with relish. At 6 P. M., pulse was 90, and of good volume. Patient passed three ounces of urine, in which there was a trace of albumen.

During the following day (28th), the pulse varied from 80 to 96, and was of good volume. A decided change was to be observed in the skin; it became less dry and dull in color, was more moist or oily, and, in fact, nearly normal. The patient took food, and getting up from her bed, set in an easy chair for an hour or so. The urine passed was of a better color and specific gravity, (1012), and had in it less than one grain of albumen to the ounce. Its quantity could not be measured.

This improved condition continued during the 29th and 30th, the specific gravity of the urine going up to 1014, and albumen remaining present to a very slight degree. On the first of October, I desired to repeat the intravenous injection, but the patient refused her consent, and I injected, hypodermically, 12 c.c. (3 *f.*ʒ) of freshly-drawn cows milk into the arm. This was rapidly absorbed, and without the slightest irritation. The next day I injected 30 (10 *f*ʒ) milk, in the same manner, without difficulty, but the effect on the condition of the patient was not, in either case, perceptible.

On the night of the second, the patient, who was a chronic opium-eater, but who had abstained from the use of the drug for

* Dr. John T. Hodgen opened the vein and introduced the canula—an operation of no little difficulty, as the blood-vessel was very small. I am much indebted to him, and also to Dr. Mudd and Mr. Dewey, for assistance in this case.

two weeks previously, succeeded in getting and taking a large dose (some four ounces) of paragoric, to which she added some laudanum,—how much I could not ascertain. At once all absorption ceased, and, gradually sinking, she died on the night of the fifth of October. I was not permitted to make an autopsy.

REMARKS.—In attempting to determine the value of intravenous injection of milk in clinical medicine and surgery, we are compelled to proceed by methods entirely empirical. Theoretically, the operation gives large promise, just as the transfusion of blood did. But it is hardly too much to say that the latter operation has not fulfilled its promise; neither does it appear that the former will do all that some have hoped for.

It was my idea, at first, to collect all the reported cases available in the current literature, and to tabulate them with the view of developing the indications for a resort to the operation, and its prospect of success. A most superficial glance, however, over the reported cases coming to my hand, convinced me of the futility of such an attempt. Such tabulation, besides eliminating the detail so essential to an appreciation of the condition of the patient operated on, would be especially guilty of those crudities with which the statistical method is charged, because the number of cases as yet reported is so small. I also hoped by a study of these cases to determine, if possible, the relative value of the operation, as a therapeutic measure, in cases of acute hemorrhage in subjects previously healthy, and in those cases where it had been resorted to for the relief of conditions characterized by blood-wasting, such, *e. g.*, as functional and organic anæmias, the hypalbuminosis of renal diseases, etc. The fifteen cases, however, details of which are at hand, afford no opportunities for any comparison, with such an object in view. This is owing to the variety of the morbid conditions for the relief of which the operation has been performed.

If the experiments of Dr. Howe (*Vide N. Y. Med. Record*, Dec. 7th, 1878, p. 444) on lower animals (dogs) are conclusive, nothing is to be expected of the operation in extensive hemorrhage; but the result may be very different in the human subject in cases of secondary hemorrhage after operation. Indeed, the cases of Dr. Gaillard Thomas, who has four recoveries in seven cases, would show as much.

But even if the injections are useless in cases of extensive hemorrhage, there still remains a large and perhaps increasing class of disorders in which they could be employed with advantage. I refer to that class of cases the chief feature of which is a deterioration of the blood. Dr. Hutchison injected fresh milk into the arm of a laborer, æt 44, suffering from extreme anæmia and debility. Three weeks after the operation the patient "seemed on the road to complete recovery." *Phil. Med. and Surg. Reporter*, March 15th, 1879, p. 230.) It is worthy of note that the transfusion of blood had previously been performed in this case without benefit. Dr. Charles T. Hunter injected five ounces of milk into the circulation of a woman, æt 32, suffering from "extreme anæmia and spinal irritation." Twenty-seven days later, six ounces were injected. "From the time of the second injection, the patient's condition improved wonderfully; she gained flesh and color, and her nervous phenomena disappeared." (*N. Y. Med. Record,* Nov. 16th, 1878, p. 397.)

Besides the case I have recorded above, I know of no other instance of the intravenous injection of milk in chronic kidney disease. In chronic parenchymatous nephritis, the most prominent blood-change is a diminution of the albumen of the plasma; those nervous symptoms that are supposed to indicate the accumulation of the waste products of the body in the circulation being, as a rule, absent, at least during the first stage. Moreover, the prognosis in this form of renal disease is not absolutely bad. My hope was to, partly at least, remedy the blood-defect in my case, and thereby gain time for further treatment. The object in performing the operation was to supply a loss caused by the local disease. I was led, furthermore, to prefer the intravenous injection of milk to the transfusion of blood, in the hope of avoiding the renal congestion that sometimes follows on the latter operation. In this I was not disappointed. The specific gravity of my patient's urine rose decidedly, but the quantity of albumen lost was not increased. Closely watching its effects, I was led to believe that the patient's life was prolonged by the operation, and this opinion was shared by Dr. Hodgen.

The fate of milk, after being commingled with the circulating blood, is a matter of practical interest. Unfortunately, our knowledge here is very limited. It appears that Donné stated, in 1844, that the milk-globules were converted into white blood-corpuscles. Researches, recently made by N. Wulfsberg, do not substantiate this assertion. He found that the white corpuscles of the blood do really increase in number, but only after they have consumed—eaten—the milk-spheres. From this, it would appear that the milk-globules have to be digested, just as well after they have been injected into the blood, as when they are taken into the stomach. How long the white corpuscles would continue to perform this function is not known. Wulfsberg was unable to prolong the life of animals by the milk-injections. Their body-weight diminished, and they died without obvious disease, and he found hemorrhagic infracts in their lungs. Hypodermic injections of fresh milk also failed to maintain life, as the animals operated on became atrophic.

Of the fate of the caseine of the injected milk, nothing, so far as I am aware, is known. In the regular order of gastro-intestinal digestion, the caseine of the milk, as is well known, is first coagulated by a special ferment in the stomach. Either this ferment is absent from the blood. or else the conditions necessary for its activity are not present. Whether the caseine is digested, as appears to be the case with the milk-globules, and if so, by what organs or tissues the proteolytic change is effected, are questions for the solution of which we must look to future research.

Wulfsberg believes that the intravenous injection of milk will not supply the place of blood-transfusion; but neither this nor the fact that the lives of animals cannot be maintained by this means, is not sufficient ground for absolutely discarding the measure as a therapeutic resource. In selected cases, and to tide over certain states imminently dangerous to life, it may yet prove of great value, and is certainly worthy of further trial at the hands of the profession, and of further research in the laboratories of the experimentalist. From both of these sources valuable information will, doubtless, shortly come; for intravenous injection of milk is getting to be quite the fashion, especially in eastern cities, and the present activity of physiological and pathological research gives no small hope from *that* direction.

